# Optimising cardioprotection during myocardial ischaemia: targeting potential intracellular pathways with glucagon-like peptide-1

**DOI:** 10.1186/1475-2840-13-12

**Published:** 2014-01-11

**Authors:** Sophie J Clarke, Liam M McCormick, David P Dutka

**Affiliations:** 1 Department of Cardiovascular Medicine, University of Cambridge, Addenbrooke’s Hospital, Cambridge, UK

**Keywords:** GLP-1, Glucagon-like peptide-1, Incretin, Ischaemia, Ischaemia-reperfusion, Cardioprotection, Myocardial metabolism, Diabetes, Percutaneous coronary intervention, Myocardial infarction

## Abstract

Coronary heart disease and type-2 diabetes are both major global health burdens associated with an increased risk of myocardial infarction (MI). Following MI, ischaemia-reperfusion injury (IRI) remains a significant contributor to myocardial injury at the cellular level. Research has focussed on identifying a strategy or intervention to minimise IRI to optimise reperfusion therapy, with the aim of delivering a superior clinical outcome. The incretin hormone glucagon-like peptide-1, already an established basis for the treatment of type-2 diabetes, also has the potential to protect against IRI. We explain the physiology and cellular processes involved in IRI, and the intracellular pathways activated by GLP-1, which could intercept IRI and deliver cardioprotection. The review also examines the current preclinical and clinical evidence for GLP-1 in cardioprotection and future directions for research as we look for an effective adjunctive treatment to minimise IRI.

## Background

Coronary heart disease is the leading global cause of death in the developed world, with myocardial infarction (MI) being associated with significant morbidity and mortality. Type-2 diabetes is another major global health burden, with prevalence continuing to increase. Compared with the general population, patients with type-2 diabetes have a significantly increased risk of MI as well as a higher risk of mortality or developing heart failure within 30 days after an event [1,2].

**Figure 1:**
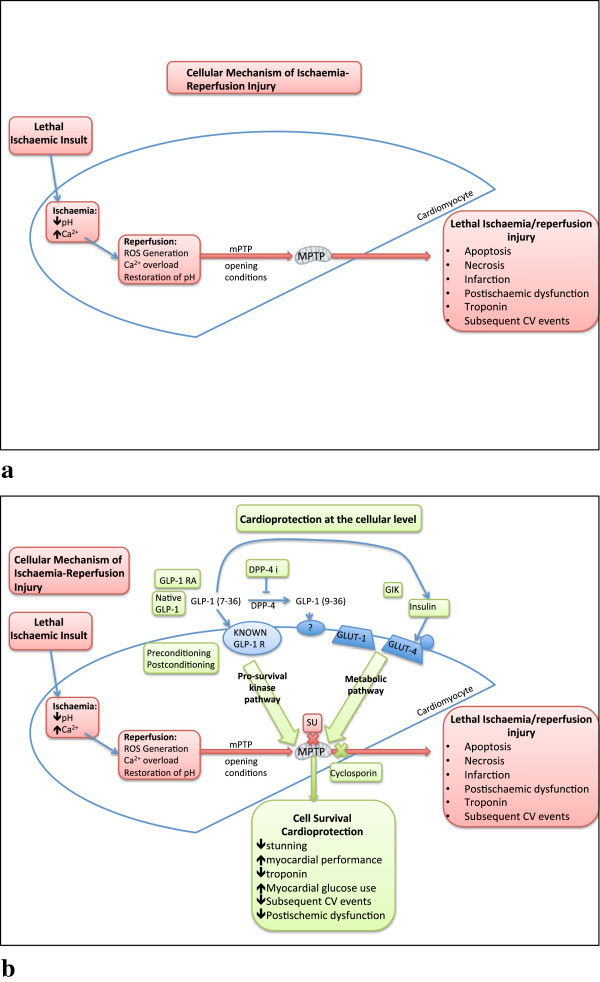
**Possible mechanisms of protection against IRI by GLP-1. (a)** Cellular mechanism of lethal ischaemic injury. **(b)** The links between the known intracellular pathways involved in IRI and the two distinct cardioprotective pathways capable of interrupting this: The pro-survival kinase pathway, and the metabolic pathway. MPTP is the intersection between the two pathways. The possible second GLP-1 receptor remains a hypothesis. Also shown are known modulators of the pathways and clinical measures of cardioprotection [9,23,24,47].

Myocardial injury following MI can be either lethal or sub-lethal, depending on the duration and severity of ischaemia endured. Sub-lethal ischaemia is often associated with reversible contractile dysfunction, although this may be prolonged due to stunning and hibernation. Stunning is transient, while hibernation is a more prolonged reduction in myocardial performance following ischaemia-reperfusion. The success of timely primary percutaneous coronary intervention (PCI) in those presenting with an acute MI has seen mortality rates fall to 6% [3], as the length of ischaemia is directly related to the final extent of infarction. The process of reperfusion itself also contributes, rapidly changing the intracellular physiological environment including activation of pathways that may induce lethal myocardial injury. Such ischaemia-reperfusion injury (IRI), with resultant apoptosis, necrosis and post-ischaemic dysfunction remains a key determinant of the final extent of infarction, degree of myocardial salvage and the overall associated morbidity and mortality of MI (illustrated in Figure 1a) [4,5].

There has been considerable interest recently in whether the IRI pathway offers a potential target to protect the myocardium beyond what can be achieved by the physical means of PCI (including thrombectomy). Important therapeutic targets include promoting myocyte glucose metabolism to enable relatively more ATP generation when oxygen supply is constrained, and activation of pro-survival kinase pathways to protect the mitochondria. Pro-survival kinase pathways are known to be activated by ischaemic conditioning; a mechanical intervention employing a series of brief, sub-lethal periods of ischaemia to protect the heart against a subsequent lethal ischaemic insult [6]. Pre-, per- and post-conditioning are performed before, during or after reperfusion respectively. The incretin hormone glucagon-like peptide-1 (GLP-1) stimulates glucose-dependent insulin release, and may also modulate the cellular processes and conditioning pathways during ischaemia-reperfusion, possibly providing cardioprotection. In this review, the evidence supporting the beneficial effects of GLP-1 on myocardial function during ischaemia-reperfusion will be discussed in the context of recent preclinical and clinical data.

### Myocardial metabolism in ischaemia-reperfusion

The primary function of the heart is to maintain the circulation. Cardiomyocytes have very limited capacity for energy storage, but a considerable demand requiring an almost continuous local synthesis of ATP [7]. To facilitate this mitochondria are particularly abundant in cardiomyocytes occupying over 30% of the normal cell volume [8]. This enables the heart to meet ATP demand with energy flowing through a series of connected, moiety-conserved cycles. However, this necessitates a continuous rich supply of oxygen and substrates to maintain optimum contractile performance [7].

Cardiomyocytes have been described as the ultimate omnivore with cells having the ability to rapidly change the metabolic substrates for ATP production depending on their availability and the energy demands of the heart. Glucose and free fatty acids (FFA) are the two most important substrates, with each inhibiting the metabolism of the other, such that the most prevalent substrate dictates cellular metabolism [7,9]. Under fasting conditions, FFAs predominate, but this is less oxygen efficient than glucose metabolism [9]. Post-prandially, increase in circulating concentrations of glucose and insulin enhance glucose uptake into the myocyte by translocation of insulin-sensitive glucose (GLUT4) transporters to the cell membrane [9,10]. Similarly, during ischaemia when oxygen and nutrient supply are restricted, there is a shift towards glucose metabolism [9]. This occurs through a poorly understood pathway that ensures the most efficient (rather than the most abundant) substrate is utilised, as cell preservation becomes more important than maintaining contractile function. Energy transfer cycles break and become more linear with anaerobic metabolism of limited glycogen stores. There is an increase in intracellular calcium, reduction in pH and accumulation of metabolic waste products [5,7]. Without rapid restoration of perfusion, these events combine to activate intracellular signalling cascades, ultimately activating the processes of cell death by apoptosis and necrosis, contributing to infarction (Figure 1a). Necrosis predominates during ischaemia if accumulation of metabolic waste and calcium is sufficient to induce osmotic swelling. At reperfusion, necrosis may become accelerated, and, if cell death signals are activated, apoptosis ensues. The result of either is an area of infarcted tissue [11].

### Intracellular pathways of cardioprotection

#### Metabolic pathway

Manipulation of the heart’s cellular energy metabolism to overcome the metabolic changes during ischaemia is an established concept in cardioprotection, as discussed above. Infusion of glucose-insulin-potassium (GIK) has been used to increase myocardial glucose uptake and glycolysis (and suppress fatty acid oxidation) protect against IRI. In preclinical studies, GIK has been protective, although transition to clinical practice has been limited. A number of studies in humans have produced variable results, potentially influenced by inconsistencies in factors such as dosage, metabolic status, glucose level and timing of administration [12]. The ‘IMMEDIATE’ study evaluated 12 hour IV GIK infusion initiated as soon as possible after the diagnosis of a possible acute coronary syndrome (ACS) before patients had been admitted to hospital. The study did not demonstrate an effect on progression of unstable angina to ACS, or 30-day mortality. There was, however, a significant reduction in the composite endpoint of cardiac arrest or in-hospital mortality, and limited imaging data showed a reduction in infarct size indicating a degree of cardioprotection [13]. The lack of definitive evidence combined with the practicalities and demands of administering insulin therapy safely have mitigated widespread adoption in clinical practice.

#### Conditioning and the pro-survival kinase pathway

Conditioning was first investigated in animals in the 1980s [14,15], and remote ischaemic preconditioning has recently been demonstrated to provide both short and long term MACE free survival benefit in humans [16]. Whilst remote ischaemic preconditioning and postconditioning have been proven effective, the mechanics and difficulty in predicting when lethal ischaemia will occur has prevented uptake in clinical practice [17].

The mechanisms of cardioprotection against IRI by conditioning have now been well established, even though the precise mechanism of activation remains unknown [4,18,19]. Derek Yellon and colleagues first described the reperfusion injury survival kinase (RISK) and survivor activating factor enhancement (SAFE) pro-survival kinase pathways and their pivotal role in the protective effects of preconditioning and postconditioning [20-22]. They involve activation of pro-survival kinases (reviewed here [23]) including phosphatidyl inositol 3 kinase (PI3K), protein kinase C (PKC), p38 mitogen-activated kinase (p38MAPK) and glycogen synthase kinase 3 beta (GSK 3β), forming a signalling cascade, converging on the mitochondria to reduce open probability of the mitochondrial permeability transition pore (mPTP) [24]. Preclinical studies support this idea, demonstrating that preventing opening of mPTP is a key step in conditioning mediated cardioprotection [25-27]. Pharmacological activation of the conditioning pathways offers promise for the future, overcoming the practical limitations of physical activation as a means of protecting against acute ischaemia-reperfusion injury [27,28].

### Mitochondrial permeability transition pore (MPTP): a key step in conditioning pathways

The mPTP is a non-selective pore located on the inner membrane of the mitochondria and, in it’s normal closed state, preserves the membrane potential and pH gradient required for the oxidative production of ATP [29]. Opening of mPTP in response to the changes in the intracellular environment during ischaemia-reperfusion is detrimental to the mitochondria and is thought to be one of the mediators of IRI though initiation of cell death by necrosis or apoptosis [29], along with other damaging effects such as calcium overload. Factors that promote opening of the mPTP in ischaemia-reperfusion include increased intracellular calcium, pH gradient, oxidative stress and depletion of intracellular ATP [29-31]. The reduction in intracellular pH during ischaemia is initially able to inhibit opening of mPTP, probably through competitive inhibition of the Ca^2+^ trigger site by protons although this has not been fully characterized [32].

During early reperfusion the pH rapidly returns to physiological levels, and this appears to be an important factor that results in opening of the mPTP at the time point of reperfusion with adverse effects on the cell. Mitochondria become uncoupled with hydrolysis rather than synthesis of ATP. Unrestrained, this will lead to the loss of ionic homeostasis and eventually necrotic cell death. Preventing this process can contribute to the efficacy of a putative cardioprotective therapy to prevent IRI, and highlights that it should ideally be administered before reperfusion [33,34].

### Cardioprotection through preventing opening of the mPTP

A number of animal models have confirmed that preventing mPTP opening can protect against IRI, with a reduction in oxidative stress and final infarct size [35]. In rabbits, this protected the heart against IRI by reducing apoptosis and necrosis both directly using a cyclosporine derivative to bind to cyclophillin-D (a component of mPTP), and indirectly by pre-conditioning [36]. Clinically it would be important to promote an environment within the ischaemic myocyte that prevents opening of the mPTP on reperfusion [21,37-40]. A number of drugs are thought to influence opening of the mPTP, and some have been studied clinically to determine whether they can protect against IRI.

Direct interaction with the mPTP by cyclosporine modifies the calcium sensitive opening of the pore and thereby maintains closure during ischaemia [11,41]. Initial clinical studies of cyclosporine in those presenting with acute myocardial infarction in whom perfusion is restored by PCI have been promising, with significant reduction in infarct size measured by cardiac biomarkers troponin I and creatine kinase. A larger randomized clinical trial is currently ongoing [42,43].

ATP-sensitive potassium (K_ATP_) channels are widely expressed, including on sarcolemma and mitochondrial membranes in cardiomyocytes [11]. The mitochondrial K_ATP_ channel is believed to play a central role in conditioning mediated cardioprotection [44]. Alongside inhibiting mPTP opening, opening of K_ATP_ is also important in protecting against IRI. It has long been appreciated that the sulphonylurea glibenclamide blocks K_ATP_ channels in the heart [11] (in addition to its therapeutic effect achieved through inhibition of the channel in pancreatic beta cell, promoting insulin release).

### Potential use of GLP-1 as a cardioprotective agent

GLP-1 is an incretin hormone, released from the L-cells of the small and large intestine in response to food intake. The intact form of the peptide, GLP-1 (7–36), has well-established metabolic actions, stimulating glucose dependent insulin release and suppressing glucagon to contribute to glucose homeostasis [45]. The metabolically active GLP-1 (7–36) is rapidly degraded by the enzyme dipeptidyl-peptidase-4 (DPP-4) to GLP-1 (9–36). Although inactive in the pancreas, it is thought that GLP-1 (9–36) may retain biological activity elsewhere, signaling either via the known GLP-1 G-protein-coupled receptor, or possibly a second unidentified receptor. Neutral endopeptidase (NEP) is also responsible for a significant proportion of GLP-1 degradation, acting at multiple cleavage sites [46], to form of further fragments including GLP-1 (28–36) and (32–36) that have been hypothesized to have potential activity at the mitochondria [47,48]. GLP-1 based therapies are now established as adjunctive treatment for type-2 diabetes, with either oral DPP-4 inhibitors increasing endogenous GLP-1 (7–36), or DPP-4 resistant GLP-1 receptor agonists being given by subcutaneous injection [45]. The resulting glucose dependent insulin release and suppression of glucagon underlies the glycaemic efficacy of these agents. Data from small studies suggest that there is also potential for a beneficial effect on the heart during ischaemia through changes in the GLP-1 pathway, and this is illustrated in Figure 1b.

**Figure 2:**
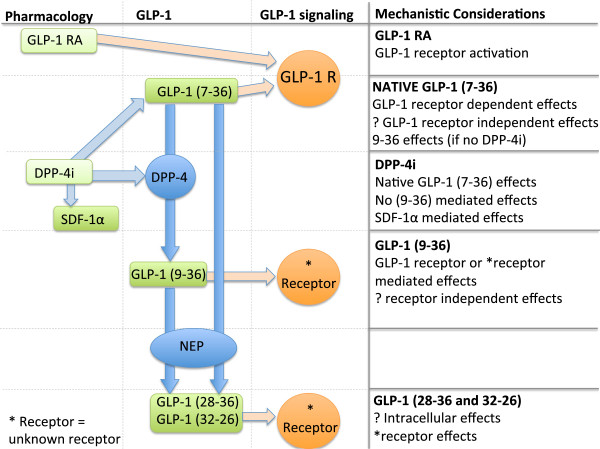
**Illustrating the different therapeutic approaches targeting the GLP-1 axis and mechanistic considerations for each**[46-48]**.** The possibilities of GLP-1 receptor independent effects and GLP-1 (28–36) and (32–36) mediated effects need validation.

The choice of GLP-1 based therapy (administration of native GLP-1 (7–36), a GLP-1 receptor agonist, or a DPP-4 inhibitor) has important mechanistic considerations in terms of which elements of the GLP-1 axis are activated. Native GLP-1 (7–36) is not clinically available, but it is metabolized into the potentially active GLP-1 (9–36) and terminal fragments. While GLP-1 receptor agonists are now widely used, they are not metabolized to GLP-1 (9–36). DPP-4 inhibitors also prevent formation of GLP-1 (9–36), and have the potential to influence other DPP-4 substrates including stromal derived factor-1α (SDF-1α) (see Figure 2). A current theory is that a chronic increase in SDF-1α may increase coronary collateralization, which is already known to improve LV diastolic dysfunction following coronary occlusion [49]. As we begin to understand which elements of the GLP-1 axis drive cardioprotection, the best approach can be identified.

### Possible mechanisms of cardioprotection with GLP-1

GLP-1 has increased myocardial glucose uptake in dogs, which although carried out in dilated cardiomyopathy, still indicates it would increase myocardial oxygen efficiency and possibly drive the metabolic pathway of protection described previously [50]. The effect of GLP-1 on myocardial glucose uptake needs to be confirmed in humans. The RISK pathway can be initiated by pharmacological manipulation of numerous cell surface receptors including G-protein coupled receptors such as the known GLP-1 receptor [21]. Preclinical evidence demonstrates that GLP-1 can activate the pro-survival kinase pathways: in murine cardiomyocytes, GLP-1 was protective, stabilizing mitochondrial membrane potential and preventing activation of apoptosis via pro-survival kinases PI3K and ERK1/2 [51]. The involvement of the GLP-1 receptor in this activation remains unclear.

In the pancreas, GLP-1 receptor binding is known to transduce cAMP-PKA signaling [52,53], and this also takes place in the heart [28,54]. DPP-4 inhibition with sitagliptin increased cAMP and PKA activity, limiting infarct size in a mouse model [55]. Most recently, GLP-1 receptor mediated cAMP signaling was observed in cardiomyocytes with downstream signaling via exchange protein activated by cAMP-2 (ePAC2) [56]. This may contribute to cardioprotection directly or via modulation of the RISK pathway [57].

Preclinical studies have shown that GLP-1 is able to reduce final infarct size. For example, exendin-4 reduced infarct size with accompanying functional improvements in rats [58]. Lixisenatide also reduced infarct size within the area at risk by 36% in rats [59]. These findings support the theory that GLP-1 can protect myocytes against lethal IRI. The infarct limiting effects of sitagliptin, vildagliptin and GLP-1 (7–36) were recently shown to be glucose dependent for the first time [60], with protection lost at glucose levels below 5 mmol/l. The suggestion that the myocardial benefits of GLP-1 might be differnet depending on the prevailing glucose concentration requires further investigation. Furthermore, the actual mechanism of benefit remains undefined and may include changes in both receptor and intracellular signaling. Recently it has been reported that high mobility group box 1 protein (HMGB1), a proposed mediator of the inflammatory response to tissue injury, may play a role in early IRI [61]. An interaction with the GLP-1 axis is supported by a study in anaesthatised rats where exendin-4 inhibited expression of HMGB1 accompanied by a reduction in infarct size [62].

### Location of the known GLP-1 receptor

The location of the known G-protein coupled receptor for GLP-1 (7–36) remains controversial, and a consensus has not yet been reached. Early work by Ban et al. showed its expression throughout the cardiovascular system in mice where a protective effect of GLP-1 (7–36) was maintained in GLP-1 receptor knockout animals [63]. It is possible therefore, that GLP-1 might also provide cardioprotection via an unknown second receptor, a potential receptor independent mechanism, or by metabolic protection. A subsequent study in isolated mouse hearts showed that the GLP-1 receptor agonist Exendin-4 conferred cardioprotection, and that GLP-1 (9–36) conferred protection to a lesser extent. The effect of GLP-1 (9–36) was again evident in GLP-1 receptor knockout mice, but was abolished by the GLP-1 receptor inverse agonist Exendin (9–39) [64], raising the possibility of a closely related second receptor. More recent work has indicated that GLP-1 receptors in the heart may be limited to only the atria and particularly the SA node, although this does not explain why improvements in LV performance and contractility are seen with GLP-1 in various other experiments [56]. Some of the actions of GLP-1 could be mediated by terminal GLP-1 fragments such as GLP-1 (28–36) [48]. These findings support the possibility of GLP-1 receptor-independent effects, but should be considered preliminary until validated by further studies [56]. Data with a highly specific antibody and confirmation with mRNA sequencing is also needed to clarify where the GLP-1 receptor is located in the heart [65].

### Haemodynamic effects of GLP-1

The potential haemodynamic effect of GLP-1 remains an area where consistent data is lacking. The question over the location of the GLP-1 receptor is central to this. Both GLP-1 (7–36) and (9–36) were vasodilators in mouse coronary arteries, at least in part via a receptor independent mechanism [63] and numerous other groups have identified a potential vasodilatory action of GLP-1 in animals [66-71]. Others have reported that GLP-1 and GLP-1 receptor agonists influence both heart rate and blood pressure in rats, potentially mediated both peripherally and centrally via the vagus nerve [72,73].

Some preliminary human data supports the preclinical findings that GLP-1 is a vasodilator, but whether this contributes to cardioprotection remains to be seen. It has been suggested that GLP-1 receptor activation with exenatide could improve myocardial blood flow in humans [74]. GLP-1 has had beneficial effects on endothelial function in humans [75,76], and a study using a dose of 1.2 pmol/kg/min produced endothelium dependent vasodilation possibly involving nitric oxide and K_ATP_ channels [77].

Heart rate increase is predictive of adverse cardiac morbidity and mortality and is therefore undesirable [78]. A meta-analysis found a small heart rate increase of 1.86 beats per minute with GLP-1 receptor agonists exenatide and liraglutide compared with placebo [79]. In a 26 week randomized controlled trial of liraglutide vs. sitagliptin, a dose dependent increase in heart rate from baseline (2.32 and 3.94 beats per minute for the 1.2 mg and 1.8 mg doses, respectively) was observed for liraglutide but not sitagliptin [80]. A single dose study found exenatide increased heart rate by 8.2 beats per minute compared with placebo accompanied by an increase in cardiac output and a reduction in total peripheral resistance [81]. So far, these observations have been limited to GLP-1 receptor agonists and a mechanism for heart rate increase is not known. Could an increase be mediated via GLP-1 receptor stimulation at the SA node, or via haemodynamic effects? Further work will help establish whether there is a clinically relevant effect.

### Evidence for acute GLP-1 mediated cardioprotection in humans

Five studies reported by our own group have examined the effects of GLP-1 (7–36) infusion on myocardial response to ischaemia in humans. Using dobutamine stress echocardiography (DSE) as a model of demand ischaemia, we have demonstrated that an infusion of GLP-1 (7–36) elicited an improvement in cardiac performance compared to controls [82]. A similar cardioprotective effect was seen when the endogenous GLP-1 plasma concentration was increased with a single dose of the DPP-4 inhibitor sitagliptin. Myocardial performance at peak stress was improved and maintained to 30 minutes, and postischaemic stunning was abolished with sitagliptin [83]. More recently, we have reported initial findings from a follow up study, where GLP-1 (7–36) administered during DSE under hyperglycaemic conditions again improved tolerance to ischaemia and abolished post-ischaemic dysfunction [84]. We conducted a pilot study during elective PCI as a model of supply ischaemia, administering GLP-1 after the first balloon occlusion finding improvements in cardiac performance [85]. The GLP-1 infusion had the expected metabolic effects, increasing plasma insulin concentration with resultant plasma glucose and FFA concentration. Initial results from the follow up study have showed GLP-1 (7–36) administered before the first balloon occlusion protected against ischaemic LV dysfunction and attenuated stunning compared to controls [86]. Throughout this work we have observed increased tolerance of the myocardium to ischaemia with attenuation of post-ischaemic LV dysfunction (i.e. reduction in stunning) with GLP-1 (7–36). The effect appears apparent in those segments of myocardium subjected to ischaemia [82].

In a randomized, controlled trial of 172 patients presenting with ST-elevation MI, an infusion of exenatide administered prior to intervention reduced final infarct size (assessed by magnetic resonance imaging) with a 15% greater salvage index compared with placebo [87]. This is suggests that GLP-1 receptor agonism can protect against IRI, with reduction in infarct size supporting the hypothesis that fewer myocytes undergo apoptosis and necrosis. Unfortunately this apparent benefit was not associated with a reduction in peak plasma troponin concentration or by a demonstrable improvement in LV function measured at 90 days following MI. It is possible that cardiac MRI will overestimate AAR by detecting transient oedema in the myocardium. The LV function at 30 days was measured at rest. Resting LV function does not demonstrate the heart’s ability to increase LV performance in response to increased demand, perhaps a more important measurement both in terms of clinical outcome and patient quality of life. An exercise or dobutamine stress test at 30 days may have been a better measure. It would be desirable to study this in future trials. Further work is also required to ascertain why exenatide apparently had no beneficial effect in patients with delayed reperfusion compared with a 30% reduction in final infarct size and 14% increase in myocardial salvage index in those where myocardial blood flow was restored promptly [87].

Our current understanding of the IRI pathways would seem to favour administration of GLP-1 before the point of reperfusion. However, two small studies in which GLP-1 was administered after reperfusion reported a beneficial effect. In addition to our own study described above, administration of a 72 hour GLP-1 (7–36) infusion after successful reperfusion following acute MI achieved both a global and regional improvement in LV function compared to control subjects [88].

These results are promising, and indicate that the protection against IRI observed in preclinical studies may also be present in humans. However, the mechanism by which GLP-1 offers protection to the ischaemic myocardium is not known. We suggest a pleiotropic mechanism, through a number of distinct, but inter-related, pathways: changes to the metabolic environment, insulin mediated effects, the pro-survival kinase pathways of cardioprotection, through cell surface receptor activation, or possibly even via receptor independent activity (Figure 1b) [18,22,23,89,90].

The common aspect of all these pathways is the ability to improve myocyte survival after an ischaemic insult, and therefore hopefully reduce the extent of infarction and preserve left ventricular (LV) function.

Various meta-analyses and pooled analyses of randomized controlled trials indicate that treatment with DPP-4 inhibitors or GLP-1 receptor agonists may reduce incidence of major adverse cardiovascular events, indicating a potentially beneficial effect of both approaches [91-95] While some of these meta-analyses indicate a lower rate of MACE events compared to comparator arms, and others indicate no difference, none of them are sufficient to decide whether or not there is a meaningful benefit. The first two prospective cardiovascular outcome studies of DPP-4 inhibitors have now reported, demonstrating no evidence of harm when used as a chronic treatment for type-2 diabetes [96,97]. The separate question of whether GLP-1 therapy can protect against acute IRI remains to be answered.

## Conclusions

The advent of primary PCI has significantly improved outcome following MI, but there is still the potential to improve myocyte salvage and mitigate IRI to further improve clinical outcome. Increased understanding of the cellular processes underlying myocardial injury, and characterisation of intracellular signalling cascades associated with myocyte loss, has identified new potential therapies to beneficially target IRI.

Preclinical studies have shown GLP-1 has the potential to reduce IRI and improve functional parameters of recovery following significant myocardial ischaemia. Early human work supports this, although the location of the GLP-1 receptor and the precise mechanism of protection afforded by GLP-1 is not yet understood.

As GLP-1 based therapies are increasingly prescribed to patients with type-2 diabetes, many of whom also have significant coronary artery disease, it will be important to continue careful surveillance to assess whether these patients are provided some degree of protection should they experience an ischaemic cardiac event. Further studies will help define whether GLP-1 is able to enhance cardiac outcome during an acute coronary syndrome, and add to understanding of the mode of action underlying any benefit.

### Limitations

This work was not performed in accordance to the PRISMA guidelines for reporting systematic reviews [98].

## Abbreviations

ACS: Acute coronary syndrome; DPP-4: Dipeptidyl-peptidase 4; DSE: Dobutamine stress echocardiography; ePAC2: Exchange protein activated by cAMP-2; FFA: Free fatty acid; GIK: Glucose-insulin-potassium; GLP-1: Glucagon-like peptide-1; GLUT4: Insulin sensitive glucose transporter 4; GSK 3β: Glycogen synthase kinase 3 beta; IRI: Ischaemia reperfusion injury; KATP: ATP-sensitive potassium channel; LV: Left ventricle; MI: Myocardial Infarction; mPTP: Mitochondrial permeability transition pore; NEP: Neutral endopeptidase; p38MAPK: p38 mitogen-activated kinase; PCI: Percutaneous coronary intervention; PI3K: Phosphatidyl inositol 3 kinase; PKC: Protein kinase C; RISK: Reperfusion injury survival kinase; SAFE: Survival activating factor enhancement.

## Competing interests

LMM and DPD declare that they have no competing interests. SJC recieves a salary from Merck Sharp and Dohme Ltd however this work was carried out independently.

## Authors’ contributions

SJC, LMM and DPD contributed equally to the preparation of this manuscript. All authors read and approved the final manuscript.
